# Stakeholder Perspectives on In-home Passive Remote Monitoring to Support Aging in Place in the Province of New Brunswick, Canada: Rapid Qualitative Investigation

**DOI:** 10.2196/31486

**Published:** 2022-05-11

**Authors:** Emily A Read, Danie A Gagnon, Lorie Donelle, Kathleen Ledoux, Grace Warner, Brad Hiebert, Ridhi Sharma

**Affiliations:** 1 Faculty of Nursing University of New Brunswick Moncton, NB Canada; 2 Faculty of Nursing University of New Brunswick Fredericton, NB Canada; 3 Faculty of Health Sciences Arthur Labatt Family School of Nursing Western University London, ON Canada; 4 Faculty of Health Sciences School of Occupational Therapy Dalhousie University Halifax, NS Canada

**Keywords:** aging in place, home care, older adults, passive remote monitoring

## Abstract

**Background:**

The province of New Brunswick (NB) has one of the oldest populations in Canada, providing an opportunity to develop and test innovative strategies to address the unique health challenges faced by older adults. Passive remote monitoring technology has the potential to support independent living among older adults. Limited research has examined the benefits of and barriers to the adoption of this technology among community-dwelling older adults.

**Objective:**

This study aimed to explore perceptions of in-home passive remote monitoring technology designed to support aging in place from the perspective of older adults, their family or friend caregivers, social workers, and government decision-makers in the province of NB, Canada.

**Methods:**

Between October 2018 and March 2020, a rapid qualitative investigation of 28 one-on-one interviews was conducted in person or via telephone. Participants included 2 home support services clients and 11 family or friend caregivers who had used passive remote monitoring technology in their homes; 8 social workers who had worked as case managers for home support services clients; and 7 individuals who were key government decision-makers in the adoption, policy development, and use of the technology in the province of NB. The interviews focused on the following topics: decision to adopt the passive remote monitoring system, barriers to adopting the passive remote monitoring system, benefits of the passive remote monitoring system, impact on client health outcomes, and privacy concerns. The interviews were audio recorded, transcribed, and analyzed by a team of 6 researchers. Data analysis was conducted using a rapid assessment process approach that included matrix analysis.

**Results:**

Participants reported that the use of the remote monitoring system allowed older adults to live at home longer and provided caregiver relief. Stakeholders were invested in meeting the home support (home care) needs of older adults. However, when it came to the use of remote monitoring, there was a lack of consensus about which clients it was well-suited for and the role that social workers should play in informing clients and caregivers about the service (role ambiguity, gatekeeping, and perceived conflicts of interest).

**Conclusions:**

Our findings highlight many benefits and challenges of the adoption of passive remote monitoring for clients, their family or friend caregivers, and public provincial health and social services systems. Passive remote monitoring is a valuable tool that can provide support to older adults and their family or friend caregivers when it is a good fit with client needs. Further work is needed in NB to increase public and social workers’ awareness of the service and its benefits.

## Introduction

### Background

Population aging is a significant demographic trend affecting countries worldwide. A recent 2020 United Nations report estimated that worldwide, the population of adults aged >65 years will double from 703 million in 2019 to 1.5 billion by 2050 [1]. The growth in the number of people living past the age of 80 years has been even more rapid and is expected to triple within the same time frame. In Canada, it is estimated that 5.5 million people will be aged ≥80 years by 2068, up from 1.6 million in 2018 [2]. Moreover, the number of centenarians grew by over 10% from 2019 to 2020, and the gap between the number of older adults and the number of children continues to widen.

This increase in human longevity is largely because of advances in medicine and public health and high population fertility rates between 1946 and 1964 (the birth of the *Baby Boomer* generation), which increased the size of this aging cohort. However, a longer life span does not necessarily mean living longer in good health [3,4]. It is well-established that at a population level, the prevalence of chronic diseases and disabilities increases with age [4]. As a result, attention has shifted from a focus on increasing life spans to healthy aging, an approach that emphasizes the quality of life and functional ability, not just living longer [4].

As older adults experience a decline in their health and functional abilities, they often require additional resources and support to safely live in their homes. In Canada, this is often achieved through a combination of publicly and privately funded home care or home support services and unpaid caregiving by friends and family. In Canada, family caregivers are estimated to support 96% of individuals receiving long-term home care [5] and are estimated to provide three-quarters of care services to older adults living at home [6], saving the Canadian health and continuing care systems an estimated US $66.5 billion annually [7].

Recently, there has been increased interest and investment in technological solutions designed to provide options for older adults to choose how and where they wish to live in their later years [8]. These technologies may also be cost-effective ways of supplementing in-person services and supporting family or friend caregivers and may ultimately prevent or delay hospitalization or institutionalization [9]. Given the current workforce shortage in long-term care across Canada [10] and the increasing number of older adults wishing to stay in their own homes, innovative technological solutions have the potential to play an important role in the lives of community-dwelling older adults and their families.

The trend toward increased use of technology in older adult care aligns with the model for geriatric care proposed by Alwan [11] >10 years ago. He envisioned a model of care enabled by technology that highlighted the potential benefits for older adults, their paid and unpaid caregivers, social and health care service providers, and health care and social systems. He imagined seamless systems that would foster client-centered care and immediate, tailored interventions based on real-time data [12]. Passive remote monitoring technologies that describe technologies embedded in the home to collect behavioral and physiological data and communicate between all stakeholders without requiring input from end users were central features of this new approach to home care, which held the promise of supporting older adults to maintain their independence for longer, delay institutionalization, and reduce costs [11,12].

Since then, much novel health monitoring and in-home technologies and systems have become available to the public, ranging from wearable smartwatches to in-home smart appliances and technology integrations that allow one to manage their home’s functionalities at the touch of their phone. Thus, there is generally more awareness and openness by the public to in-home technologies that can assist people with activities of daily living and entertainment [13]. However, passive remote monitoring systems tailored to the needs of older adults with increasing home care or home support needs and their caregivers seem to be less widely used, in part because of concerns about personal privacy by multiple stakeholders, costs, and uncertainty about their effectiveness [14].

### Home Support Services for Older Adults in New Brunswick

In New Brunswick (NB), Canada, many older adults are eligible to receive publicly funded home care and home support services [15]. In this provincial context, *home support services* refer to nonmedical services such as assistance with activities of daily living, respite care, and help with shopping or errands. The province’s Department of Social Development is responsible for funding and managing home support services for older adults who are eligible to receive them, whereas a variety of third-party companies or individuals deliver these services. In contrast, *home care* refers to services provided by regulated health care professionals (eg, registered nurses and occupational therapists). Home care is funded by the province’s Department of Health [16]. In terms of funding, individuals can pay out of pocket for either type of service if they are not eligible to receive publicly funded services or if they wish to supplement the services they receive. In this study, we focus on an in-home passive remote monitoring system that is available as part of the suite of publicly funded home support services offered by the NB Department of Social Development.

### The Passive Remote Monitoring Service

CareLink Advantage is a private company that operates in several Canadian provinces. Their service was initially developed by adapting home security system technology to address the specific needs of community-dwelling older adults requiring increasing levels of support to maintain their independence. The system was designed so that it can be tailored to client needs, offering a client-centered solution to home support needs. In terms of the physical system, clients can choose a combination of passive monitoring devices for their home system, including motion detectors, bed and chair sensors, medication adherence monitors, and motion-activated video cameras. Once installed, the system allows for ongoing passive remote monitoring to occur. First, behavioral norms are established for clients. Then parameters are set to alert a family or friend caregiver by phone or SMS text message when unusual behavior such as wandering or mismanagement of medication has been detected [17]. These real-time notifications allow the caregiver to check on the client and intervene if necessary. In addition, family or friend caregivers can log into a secure portal where they can see client data such as sensor activity and 15-second video clips of the outside entrance of the home, allowing them to see changes over time [17].

Since 2008, CareLink Advantage has been included in the suite of services funded by the NB Department of Social Development for older adults in the province who are eligible to receive publicly funded home support services [18]. However, despite older adults making up >20% of the province’s population, adoption has been extremely low, with <100 clients having used the service over the past 12 years (McDonald, personal communication, 2020). It is unclear why adoption has been so low. Therefore, the purpose of this study was to understand the experiences and perspectives of key stakeholders in NB regarding the adoption of CareLink Advantage.

## Methods

### Overview

We conducted a qualitative study using semistructured interviews that were analyzed using a rapid assessment process [19] to generate a preliminary understanding of the experiences and opinions of key stakeholders in the province regarding the remote monitoring system. This rapid assessment process is an intensive, team-based ethnographic approach to qualitative data analysis that uses triangulation, iterative data collection, and iterative data analysis to quickly gain *the insider’s perspective*, which informs future data collection and analysis [19]. Project timelines can be as short as 4 to 5 days, with at least two researchers involved; however, the study becomes more robust with more researchers and time spent on data collection and analysis [20]. This process is also noted to benefit from a diverse team of researchers performing research, where the combination of experience and knowledge acts as a substitute for the time spent in the field.

This rapid assessment process was an ideal choice of methodology for this study as it suited the need to efficiently analyze a large and complex data set containing 28 qualitative interviews with >35 hours of transcripts, spanning >4 different data subsets representing clients, their family or friend caregivers, social workers, and decision-makers. This approach facilitated the process of comparing data subsets and gaining a better understanding of how the remote monitoring system is perceived across the 4 key stakeholder groups. The quick turnaround of this analysis generated the timely findings needed to inform 2 other related and concurrent projects evaluating the use of the remote monitoring system in 2 other Canadian provinces. Using this method also leveraged the wide range of expertise and experience represented by a core team of 6 researchers and 3 additional participants throughout the lifetime of the project.

### Participants

To obtain a rich data set that captured a range of perspectives on passive remote monitoring technology, we sought participants from 4 key stakeholder groups using a combination of purposive, snowball, and convenience sampling. The target stakeholders included (1) older adults aged ≥55 years who used the remote monitoring system in the province with or without subsidy from the Department of Social Development, (2) family or friend caregivers with experience using the passive remote monitoring system in the past or present, (3) social workers with experience working as case managers for older adults receiving home support services from the Department of Social Development, and (4) individuals who were engaged in the initial adoption and ongoing administration of the passive remote monitoring system as part of the range of home support services covered by the Department of Social Development (ie, government decision-makers). Social workers were eligible to participate regardless of whether any of their clients had used the technology. Clients and family or friend caregivers who did not have experience using the system were not eligible to participate in the study.

Participants were recruited using several approaches. Eligible clients and family or friend caregivers were identified and contacted by the Department of Social Development and CareLink Advantage to inform them about the study and invite them to contact the research team if they were interested in participating. This indirect approach was used to protect client privacy. The Department of Social Development also sent out a recruitment email internally to the staff, including social workers who worked as case managers for older adults. In addition, the research team used snowball sampling [21] by asking interview participants at the end of their interviews to inform people in their social circles about the study if they were eligible. Finally, key stakeholders who had been involved in the initial adoption of the passive remote monitoring system in this province but were now retired were personally invited directly by the research team to participate in interviews. All participants received a letter of information about the study and provided written informed consent before partaking in the interviews.

### Data Collection

A series of in-person or telephonic semistructured qualitative interviews were conducted using an interview guide. The questions were designed to explore participants’ experience using (clients or caregivers), recommending, or implementing (social workers or government decision-makers) the remote monitoring system. Some questions specifically focused on the implementation process at the public and individual levels. Other questions were included to shed light on the issues of client information sharing, storage, and privacy, as this was a common barrier anecdotally reported to be hindering the adoption of the passive remote monitoring system in the province. The questions led each participant to discuss their experience with how the remote monitoring system supported the client’s ability to delay or eliminate the option of leaving their home for long-term care.

### Data Analysis

Following the steps outlined by Hamilton and Finley [22], a team of 6 researchers collaboratively developed a data extraction template based on semistructured interview guides used for data collection. The data analysis process was tested and refined by independently applying it to 2 interview transcripts, reviewing the results, and collaboratively refining the process. Next, the researchers were split into 2 teams of 3 researchers to analyze the interviews. One team analyzed client and caregiver (n=13) interviews, and the second team analyzed social worker and government decision-maker (n=15) interviews. Each researcher independently reviewed the assigned transcripts and met with their team (3/6, 50%) to compare and discuss their findings. Then the 2 teams (6/6, 100%) met to compare, discuss, and summarize the findings into a matrix. Team members had varying levels of personal engagement with digital technologies to support the caregiving of older adults, and these personal experiences were used at times to delve deeper into a particular quote or theme that was emerging. Through dialog between team members, we discussed the differences in our analysis and arrived at a shared understanding of the data. To ensure trustworthiness, the following strategies were used throughout the data analysis process: peer debriefing, investigator triangulation, iterative analysis, and maintenance of an audit trail [21,23].

### Ethics Approval

This study was approved by the Research Ethics Board at the University of New Brunswick (REB#: 2017-057).

## Results

### Participants

Participants included 2 home support services clients and 11 family or friend caregivers who had used passive remote monitoring technology in their homes; 8 social workers who had worked as case managers for home support services clients; and 7 individuals who were key government decision-makers in the adoption, policies, and use of the technology in the province of NB. Caregiver participants were not necessarily associated with the 2 client participants.

On the basis of the information shared during the interviews, participants had the following characteristics: the 2 client participants lived in close proximity to their caregivers and had a comprehensive setup that included cameras; medication dispensers; and passive sensors on the bed, refrigerator, and exits of the home (Table 1). Of the 11 caregiver participants, 9 (82%) were adult children caring for parents, 1 (9%) was caring for a relative or sibling, and 1 (9%) was caring for both a parent and a sibling. Of the 11 caregivers, 1 (9%) lived with the client, 2 (18%) lived outside of the province, and 8 (73%) lived in close proximity to the client. Approximately 55% (6/11) of caregivers reported their loved ones having a camera in their home. Of the 8 social worker participants, 6 (75%) had received a 1-hour training session from either the passive remote monitoring company or the local contractor hired to service the passive remote monitoring company’s clients. None of the participants confirmed that they had participated in the pilot study. In the decision-maker group, of the 7 participants, 3 (43%) were former social workers who had clients using the remote monitoring system, and 2 (29%) were hired by a community organization funded by the province to pilot the remote monitoring system in the province.

**Table 1:** Remote monitoring component use and paid in-person care across client and family or friend caregiver data subsets.

Interviewer code	Participant type	Remote monitoring system components				
		Bed sensor	Door or room sensor	Medication adherence	Cameras	Paid in-person care
1006_01	Patient	✓	✓	✓	✓	
1009_01	Patient	✓	✓		✓	
1008_01	Caregiver	✓			✓	
1010_01	Caregiver	✓	✓	✓	✓	
1014_01	Caregiver	✓	✓		✓	✓
1015_01	Caregiver	✓	✓		✓	
1016_01	Caregiver	✓	✓			
1018_01	Caregiver	✓	✓			
1020_01	Caregiver	✓	✓		✓	
1022_01	Caregiver		✓		✓	
1024_01	Caregiver	✓		✓		
1025_01	Caregiver	✓	✓			✓
1027_01	Caregiver	✓	✓			✓

### Key Messages

The summary matrix (Multimedia Appendix 1) provides the key messages by each participant group for the categories included in the data analysis template. The categories were (1) the decision to adopt the passive remote monitoring system, (2) barriers to adopting the passive remote monitoring system, (3) benefits of the passive remote monitoring system, (4) impact on client health outcomes, and (5) privacy concerns.

#### The Decision to Adopt the Passive Remote Monitoring System

##### Decision-Maker Adoption

Approximately 29% (2/7) of the decision-maker participants in our study were directly involved in bringing the remote monitoring system to the province. In their interviews, they recounted how they had first learned about the technology at a conference in Ontario and then worked with the Department of Social Development to spearhead a pilot that led to CareLink Advantage being included in the basket of services available to older home support clients:

...as we were touring exhibits, we saw CareLink and we had never heard of it and so the guy showed us how on his Blackberry he could see how his mother who had dementia was managing with her meds and her mobility in her apartment. She was miles away and it kind of blew us away, we never thought of that and so he explained how the technology works with basic home security apparatus and cameras and with camera positions specifically over the medications so he could tell whether she had taken her pills today or not and her whole system of alarms on doors, alarms on the bathroom and elsewhere and we looked at that. We thought my goodness, that has great applications for New Brunswick and so we came back and brought the publicity material to the Department of Social Development who agreed to a pilot project. I think there were several nursing homes involved and we had clients scattered around and so on the success of the pilot project, the department agreed to make it a benefit for persons dealing with parents at home. We could see right away that it had enormous potential

Participant 1029, lines 7-27

Both decision-maker participants indicated that there were few barriers to government adoption. However, they noted a lack of awareness of the service. For example, participant 1029 stated the following:

Every family that I’ve asked, my first question is did anybody explain this technology to you and they say no...I ask them, social workers or people in the field, what do you know about CareLink and they just draw a blank because they don’t know.

Participant 1029, lines 136-141

##### Client Adoption

It seemed to be well understood across all participant groups that the technology had to be considered a good fit for the needs of the client and their family or friend caregiver by all parties involved in deciding whether to install remote monitoring devices (social worker, client, and family or friend caregiver).

Social workers played a key role in determining who was a good fit for the service based on their assessment and circumstances and shared information about the system only when they thought it would be appropriate. The importance of a good match between client needs and CareLink Advantage was highlighted by several social worker participants. As one of the participants explained, introducing CareLink Advantage to a client involved using “...their own kind of understanding of what the technology is to share with the client when they feel it’s something that might meet the need of a client so they wouldn’t be exploring it in every situation, just when they think that there’s an appropriate use for it” [Participant 1017, lines 314-319]. Another stated the following:

...not everyone receives the information because it wouldn’t be appropriate for everyone to receive it either and that’s part of the social worker’s role when they’re developing their case plan and talking to families.

Participant 0206, lines 132-135

Although the fit was identified as being very important, social workers were divided on their perceptions of which clients they should recommend passive remote monitoring to. For example, the geographical distance between clients and their family or friend caregivers was interpreted differently. One social worker stated the following:

...they need some form of supervision...typically, those are the people who are living independently who have family nearby who CareLink have an option for.

Participant 1004, lines 40-51

In contrast, a decision-maker who used to be a social worker shared stories of how the service had been extremely helpful to a caregiver who lived in another country:

...after we installed that [monitoring devices], she came twice or three times a year and it was less stressful for her. She felt like she knew what was going on. She felt like she was able to make sure that her mom was treated well. Like she felt like she was there, so huge impact.

Participant 1019, lines 699-704

Cognitive status and wandering behaviors were also considered by the social workers when making their recommendations. Some viewed the technology as an excellent service for clients who needed high levels of supervision and felt the technology could delay or avoid residential care. As one of the social workers explained, CareLink Advantage can be a helpful service for clients experiencing memory loss and their families:

Family members are concerned because they forget and most of the time too it could be like security issues like they’re forgetting like the stove on. They might go for a walk and don’t remember where they live. They could lose themselves like they’re wandering.

Participant 1023, lines 276-280

This participant stated that in these situations, “...[CareLink Advantage] helps certainly because it reassures everybody. It shows either the need for a placement or it shows either that the person is still able to stay in her house” [Participant 1023, lines 415-417]. Another social worker emphasized the following:

...supervision is a big thing when a client has Alzheimer’s or dementia and...families often want to keep their loved one home [as] long as possible. So, the more supervision we have in the home, the more possible it is to do that. And that’s what I think CareLink can do, is kind of provide more of that supervision piece without giving up too much privacy.

Participant 1003, lines 233-236

In contrast, some social workers felt that clients with substantial cognitive impairments and a high propensity to wander were too risky for passive remote monitoring as they always needed a person with them or at least the caregiver to continually monitor their electronic devices for any notifications. For example, one of the social workers stated that CareLink is “Not for somebody that is at risk of wandering, but it’s good when they are kind of in their early stages and then they are kind of in that gray area” (Participant 1007, lines 36-38). Another emphasized the importance of having family or friend caregiver support for the service to be useful:

Certainly if people are able to stay in their home like longer, but it has its limits too. Like one time I had one client she had Lifeline, CareLink, but the family/friend caregiver was not checking his phone or his computer so he’d get nothing.

Participant 1023, lines 508-514

Family or friend caregivers were also identified by almost all participants as playing a key role in deciding to adopt the remote monitoring system. This was in part because of the requirement of having a designated person to receive system notifications if anything unusual should happen. It was also reported that caregivers played a stronger decision-making role in selecting home support service options when older adults had cognitive impairment and were less able or unable to make their own decisions. Whenever possible, clients were directly involved in the decision about adopting the passive remote monitoring system. The 2 clients who participated in our study reported that they were selected to have CareLink Advantage and agreed to have it. One client shared the following:

...they didn’t ask us if we was interested, they come in and said we were selected. And wanted to know if we’d like to have it, and I thought, Oh, this is a Godsend. This is wonderful. So they spent over a half day here, and put things together, putting things up and I was so happy.

Participant 1009-01, lines 60-63

#### Barriers to Adopting CareLink Advantage

Perceived barriers to the adoption of CareLink Advantage included a lack of awareness and knowledge of the service, preference for traditional or familiar home support services, additional caregiving responsibility required, hesitancy to promote a service offered by a private company, costs, privacy, and language.

Lack of awareness and knowledge of the service was identified as the primary barrier to adoption. In particular, social workers felt that the passive remote monitoring system was not promoted or visible enough in the province, making it difficult for clients and caregivers to understand what it was and how it worked:

I mean they gave us pamphlets as well to pass out to clients...but it’s still a newer service to introduce to clients. So you kind of have to keep reminding yourself that it exists.

Participant 1012, lines 35-37

Some social workers expressed an interest in learning more about the technology so that they would feel more comfortable explaining the service, whereas others wanted informational pamphlets to hand out to clients. There was also sentiment by some social workers that it was not their job to promote the passive remote monitoring system or explain it to people:

...it’s my decision to offer it and put their decision...forward it if they want it, but making the decision to have it has to come from the client.

Participant 1012, lines 182-184

Decision-makers felt that the biggest barrier to adoption was communication between social workers and caregivers or clients about the service. They identified several structural factors that influenced social workers’ knowledge about the passive remote monitoring system and their ability to discuss it with their clients and caregivers. Social workers’ gaps in knowledge about available services were thought to be the result of a combination of high turnover and insufficient orientation to the services. For example, one of the decision-makers shared the following:

The staff in the regions don’t get a formal prescribed orientation or training program, they just kind of pluck [them] in the job and they learn, they pickup stuff from the person that was previously in that job. Whatever they pickup they pickup pretty informally and so if the person that occupied that particular social work position before knew nothing about CareLink Advantage, guess what, the new person coming in is gonna know nothing about CareLink Advantage either.

Participant 1029-01, lines l178-1183

In addition, decision-makers felt that because of the social workers’ demanding workloads, they had little time to learn more about the passive remote monitoring system or take on the responsibility of teaching or guiding caregivers about the technology:

...there is pressure there to put in plans that you know will be successful because for you to go back in and see them every 3 months because things are unfolding, can be a challenge when you have a number of clients that you’re managing...so it’s finding that balance of...what do I know is successful and how do I get that setup so that people aren’t knocking on my door every minute.

Participant 1017, lines 716-721

There was a sense that social workers, clients, and caregivers were apprehensive about the technology and preferred more traditional services with which they were familiar, especially when they were in crisis and feeling overwhelmed. For example, one of the decision-makers shared the following:

...some people’s misunderstanding as to the benefits of the technology is also a challenge. People still really push for needing the hands-on care then they feel they’re not doing anything if there isn’t someone physically there doing the care.

Participant 1017, lines 227-231

This was supported by statements from social workers such as the following:

But I think that a lot of the reason why I don’t use it as much, for one, I don’t offer it to every family because sometimes a person is needed and CareLink would not be enough to meet the need or address the concern.

Participant 1004, lines 122-126

Another social worker noted that when in crisis, caregivers often feel overwhelmed and unable to take on a new challenge or responsibility and see how the passive remote monitoring system can help them. They said the following:

The caregiver is saying like I’m stressed, I’m burnt out, I just don’t have the capacity...she’s getting up at night and I know it’s happening five times a night, I need someone to be there to manage that, not me, that kind of thing. So, I think there’s probably some ways you could you know, if we were creative about the problem-solving we probably could make it work, but by that time the caregivers are like turned off.

Participant 1017, lines 390-393

Decision-makers from the NB Department of Social Development felt that some social workers seemed to view discussing CareLink Advantage with clients as a conflict of interest as the service is run by a private business. One of the participants explained the following:

...it was unclear to them what their role was:...they’re looking to the social worker to help explain that and kind of promote that when that’s not really their role, right...Because...the expectation of the social workers...give the client the options of what’s available to them.

Participant 0206, lines 97-98 and 102-103

Caregivers perceived the cost of the internet as a barrier to using the system, although it was only required if video cameras were desired in the home. Social workers also raised concerns about cost, internet access, and potential power outages, particularly in rural areas of the province. Caregivers also reported pushbacks from home support services agencies related to personal care workers who had privacy concerns if they were monitored when they came into the home. Installation services not offered in the client’s preferred language were also identified as a potential barrier. Finally, client pride was also identified as a potential barrier, with one of the participants noting the following:

...it’s hard for a lot of people to admit that they may need that level of monitoring, right?

Participant 0206, line 151

#### Benefits of CareLink Advantage

Benefits for caregivers and clients were identified by all participant groups. Interestingly, decision-makers felt that the main benefit of the passive remote monitoring system was to caregivers who require support, with one explaining the following:

...you see it more as a support for the caregiver to kind of give them that relief and that sense of security of what’s going on in the home.

Participant 1017, lines 1202-1204

For clients, the main benefits identified were increased peace of mind and increased access to services when needed. One client noted the following:

...with this in your home, it’s more-or-less lifesaving... If something happens, it goes directly to their home, or television or cellphone, whatever it’s on.

Participant 1009, lines 103-107

Caregivers also reported that clients felt safer and that they themselves experienced a sense of relief and reassurance that their friend or family member was safe. One caregiver stated that with the system in place,

...it was safe...for us to go to work knowing that if she opened the door somebody will notice...and we would react, we would go or call first and then if she didn’t answer well, we got in the car and go see where she was.

Participant 1027, lines 218-220

In addition, caregivers reported feeling less stress, greater peace of mind, and better sleep, and some were able to take vacations, knowing that they would be alerted if anything was wrong. One of the caregivers stated that the remote monitoring system provided a strong sense of relief to her and her siblings:

It took away so much stress for me, it was unbelievable, I was at my wit’s end worrying all the time. It was such a big, big relief for me and my sister...because she’s so far away and she’s so guilty that she’s not here to help. She knew the burden was on me, but...she could see how, you know, what my mother was doing through the sensors.

Participant 1016, lines 252-258

Another highlighted the sense of peace and relief from worry that the system provided:

...it gives you a good peace of mind that everything is under control and you don’t need to worry...

Participant 1025, line 547

A third caregiver provided an example of how they were able to remain connected and reassured when apart, even on vacation:

...So, we’re on vacation you can just click in, and you can see him there and if we wanted to talk to him on the phone you clicked, he was in his chair in the kitchen close to the phone well then, we would call, and you’d know he could reach the phone.

Participant 1020, lines 365-370

In addition to providing benefits to clients and caregivers separately, social workers mentioned that the passive remote monitoring system contributed to better relationships between caregivers and clients. For instance, one of the participants shared that having CareLink Advantage allowed caregivers to focus less on the older adult’s health. They stated the following:

Mostly what you hear are the caregivers, right, that they feel much more at ease and more comfortable, they can start to have different conversations with their, you know, often it’s their parents you know instead of you know, did you take your medications or whatever they know, right, so it can help with that.

Participant 0206-01, lines 347-350

Another benefit perceived by caregivers was the ability to monitor the care provided by home support workers. For example, one of the caregivers shared that having the passive remote monitoring technology in the home provided confirmation that care was being provided as expected:

The expected time for them usually to arrive is usually between 9:30 and 10:00 in the morning and I could see you know, the door was open and I would get a ding on my thing to indicate the door was open or had been opened. Through the motion I could see, through the motion sensors so graph bars that there was activity, oh, now they’re in the bathroom. She’s getting a shower so I knew that she was getting the services from the personal care worker, they were doing what they were supposed to be doing as regards to showers...

Participant 1016, lines 158-165

Another caregiver shared that they did not trust home support workers because of negative experiences that they had in the past:

She was supposed to give him a shower she never did. She went out seven times outside to smoke, seven times in three hours. That’s not normal.

Participant 1024, line 854

This participant stated that the system provided reassurance and suggested that it should be used more widely to prevent abuse:

It was reassuring for me. That’s why I want it out there more. I want the social worker to push it because I want to see it more and you see where the abuse is being made.

Participant 1024, lines 852-853

Decision-makers and social workers also identified cost savings to provincial health and long-term care systems, as well as to families, as a key benefit, as the passive remote monitoring system is less expensive than in-person care. One of the social workers added that it might help address the current workforce shortage.

#### Impact of CareLink Advantage on Client Health Outcomes

Caregivers reported no changes in health care use, whereas social workers and decision-makers stated that there was no formal process to evaluate the impact of the passive remote monitoring system on client health outcomes. However, all 4 groups reported that clients using the passive remote monitoring system were able to stay in their homes longer or completely avoid going to a nursing home:

...it kept her in her home until she passed away...So it was just the best thing that could ever happen, it was very very good, positive and the people were so, they were just so wonderful to work with.

Participant 1015, lines 61-64

A social worker commented on the health outcomes related to staying at home longer:

...I see often-times a lot of clients need to go to special care homes or nursing homes that do have 24-hour supervisions, but those transitions can be really, really tough. And sometimes clients’ health deteriorate with those transitions leaving home and being in a new environment and it’s hard to adapt to that...in terms of possibly keeping them home longer and what is affecting you know their emotional health, maybe in that regard [it is beneficial].

Participant 1012, lines 257-265

Decision-makers highlighted that it supported client choice:

...it’s giving them other options and it’s offering them the ability to stay home longer...We want to be able to keep seniors in their home as long as it’s possible so I think with CareLink that’s what it gave us. It gave us more options to be able to do that.

Participant 1019, lines 375-379

Clients also reported increased accessibility to emergency services when required and increased peace of mind and sense of security. For example, one of the clients shared their experience using the system to access emergency services when they fell:

...I came to on the floor, between the bathroom and the bedroom, and I pushed the button then and I guess it must, I can’t remember whether [name removed] was up here at the time or they called him and told him. But they had an ambulance come and they [paramedics] took me to the hospital, because they call the ambulance for you.

Participant 1009, lines 108-112

Caregivers stated that the passive remote monitoring system allowed them to keep track of evolving care needs such as increased supervision, maintaining client routines, ensuring adequate nutrition, and assisting with adherence, adjustments to treatment, and medication protocols through observation of changes in behaviors such as increased sleep in older adults. One of the participants shared that having CareLink Advantage in her mother’s home allowed her to monitor her pain:

She was bed, not to say bedridden, but she was always laying in bed for the longest times whether through boredom or through just, she told me a lot of times she’s just tired, arthritic pain. We were able to monitor and watch that. It then became a concern, it actually became a concern to us because we noticed that she was in bed a lot, more so than we would ever have thought so that became an issue for us. It raised to the point that when I told the doctor about how long she was staying in bed, well take a look at her meds and stuff and we started actually adjusting her meds.

Participant 1016, lines 88-95

Similarly, social workers identified CareLink Advantage as facilitating the tracking of evolving client care needs. They emphasized that although the passive remote monitoring system helped keep clients home longer, at some point, institutional placement was often unavoidable when care needs were beyond what home support services could offer.

#### Privacy Concerns

Interestingly, clients themselves had few personal privacy concerns about the monitoring, although they acknowledged that caregivers and visitors coming into their homes might feel it was an intrusion. One acknowledged that the passive remote monitoring system might provide more privacy than having personal support workers coming into the home:

Well, I guess, it does make me feel safer...There was a time when I didn’t, when we had certain caretakers in here with the key to everybody’s door...

Participant 1006, line 276

This view was also expressed by a decision-maker who indicated technology was less invasive and disruptive than having multiple home support workers entering the home:

...in a way it may be invading their privacy in a way, but it’s less invasive than if you have a person in your home like everyday.

Participant 1021, lines 352-353

On the other hand, family or friend caregivers were concerned about the privacy of the client but felt that this was outweighed by the additional sense of security and honoring their wish to stay in their own home. A family or friend caregiver discussed choosing monitoring devices that would have less effect on her family member’s privacy:

...let’s start off with the door and the mattress pad and let’s just start slowly... She’s only alone like half an hour in the morning and two in the afternoon, but when we leave for lunch we know she goes to bed from one to three.

Participant 1023, lines 314-318

Consistent with the views of the clients in our study, some caregivers reported that clients did not mind the cameras and sensors and did not have objections related to privacy. They also identified the privacy of paid caregivers coming into the home as a concern.

Social workers’ concerns about privacy were centered on the use of technology, which conflicts with provincial privacy legislation. These concerns focused mainly on the video components of the system:

I think the idea of cameras is really scary to a lot of people...most seniors they want to be as independent as they can be and the idea of someone checking in on them, or being notified, you know, is taking away their independence...I think the idea of cameras can be really off putting and viewed as really invasive...I mean as soon as cameras are mentioned, their eyes go wide. You know they are shaking their head and they just have no interest at all.

Participant 1012, lines 290-296

They also questioned the privacy of care providers or visitors who were not aware of the presence of cameras and what information should be available to alert them:

...the cameras, in the spare room or something just in case they open the client’s cameras and...went in to change from their one set of change to their work clothes or whatever the case may be, or just for the person general knowledge that there are cameras and that there is someone looking in.

Participant 1012, lines 400-407

The perceived impact of monitoring devices on privacy was viewed as a barrier to the adoption of passive remote monitoring systems in the province. The option of having cameras in the home was perceived by the decision-makers as being particularly concerning to the social workers, a view that was supported by the interviews with social workers. Decision-makers perceived social workers as focusing too much on their own concerns and not enough on the benefits of the system. Finally, the decision-makers also reported that more stringent data collection, storage, and management policies had been applied at the Department of Social Development since the pilot and the need to keep up with evolving provincial and federal policies on health data security.

The decision-maker group perceived that social workers had a strong ethical lens and were committed to protecting the privacy of their clients. However, they felt that privacy issues needed to be weighed against safety, and if the home support services client were to be admitted to a long-term care facility, their privacy would be compromised even more:

There have been some well positioned persons of influence who are really, who expressed very strong feelings on the privacy thing...our response to that has always been look, the elder is living at home with dementia and cognition may be seriously compromised anyway and their son who is the power of attorney and responsible for mama’s care signs off on this thing, stop worrying about the privacy issues because at the end of the day what we have to be more concerned about is the safety of your old mom...I think the response for that is, you know, you got to apply common sense, good reason and you got to make sure that the care of mom is top priority.

Participant 1029, lines 224-236

Another decision-maker highlighted the heightened concern over privacy when cameras were in the home and felt that it was unwarranted, given that regardless of the presence of a camera, older adults have more privacy at home than they would when living in a nursing home:

So staff perception of the cameras was heightened and I believe that was the biggest barrier to implementing it...I said to them you know, if you believe that somebody who leaves their home early and goes to a nursing home is going to have more privacy than you would have in your own home with cameras that are only being viewed by family members, then you need to think again.

Participant 1030, lines 63-71

## Discussion

### Principal Findings

Overall, it was clear that all stakeholders shared a common goal of helping meet the individual needs of older adults who require support services to live safely in their own homes using a client-centered approach. However, when it came to the use of the CareLink Advantage remote monitoring service as a tool to help meet these needs, there was a lack of consensus about which clients it was well-suited for and the role social workers should play in informing clients and caregivers about the service. Our findings highlighted many benefits of the passive remote monitoring service for clients, their family or friend caregivers, and public provincial health and social services systems, as well as the challenges associated with adopting novel technology that people are unfamiliar with or uncertain about. To our knowledge, this is one of the first studies to examine an in-home remote monitoring system for older adults by triangulating data from 4 diverse stakeholder groups. Thus, our research contributions are 2-fold. First, our findings provide insights that advance the understanding of the implementation and use of in-home remote monitoring systems in older adults’ homes. Second, this study provides a useful example of a rapid methodological approach that can be replicated by others.

### Staying Home Longer

A key finding was that all participant groups reported that the remote monitoring system allowed clients to live at home longer than they would have been able to without the technology. To date, limited research has examined the impact of in-home remote monitoring systems on older adults’ ability to live at home longer. A recent scoping review [24] identified only 14 studies published before February 2019 that examined outcomes related to having this type of technology in the homes of community-dwelling older adults. Although a wide range of outcomes was assessed in these studies, none of them tested the impact of passive remote monitoring on the length of time older adults were able to live at home or time until institutionalization was required. One qualitative study did find that older adults reported a strong desire to age in place and saw passive remote monitoring technology as a tool to make that happen [25]. Although our study provides preliminary evidence that suggests that in-home passive remote monitoring technology may enable older adults to live at home longer, further research with robust quantitative designs is needed to test this relationship.

More recently, Pais et al [26] conducted a 12-month observational study in Switzerland to evaluate the useability, functionality, and effects of an in-home monitoring system comprising a combination of wearable and passive monitors on older adults, their family caregivers, and home care nurses. Consistent with our findings, the study by Pais et al [26] found that most older adults, family caregivers, and nurses perceived that the monitoring system helped older adults stay at home longer. Similarly, a recent systematic review of stakeholder perspectives on technology use among community-dwelling older adults with dementia found that the perceived potential for technologies to allow them to stay in their own homes and avoid or delay institutionalization was an important facilitator of technology adoption among older adults with dementia [27]. They also found that family or friend caregivers had positive perceptions of technologies with the potential to enhance the independence and quality of life of people with dementia [27].

Our findings contribute to the growing evidence that suggests that providing older adults with the option to live at home is important and that in-home technologies are perceived as a means through which to achieve this desired outcome. Moreover, our qualitative findings, along with those of others, support the need for stronger empirical evidence linking in-home technology interventions to staying at home longer and delaying or avoiding institutionalization.

### Caregiver Relief

Another key finding was that the remote monitoring service provided valuable benefits for family or friend caregivers of older adults. In discussing the benefits of remote monitoring technology to support aging in place, many social workers and policy makers mentioned the family or friend caregiver’s need for support. The role of a family or friend caregiver is to fulfill an increasing demand for home-based care, precipitated by an aging population and governments promoting policies to alleviate the pressures on the health and continuing care systems [28]. This role can be unsustainable for unpaid family or friend caregivers who juggle paid work in addition to maintaining the care recipient’s needs; it is reported that family or friend caregivers often have no choice to reduce or leave paid work to maintain the needs of the person they care for [20]. Unpaid caregiving can also have negative consequences on relationships between the caregiver and the care recipient, other family members, and across wider social circles [29]. Physical injuries and burnout are also common outcomes of unpaid caregiving [12]. These are some of the negative social, financial, and health repercussions associated with the unpaid family or friend caregiver role [28].

In our study, all groups recognized the impact of the passive remote monitoring system on improving the family or friend caregiver’s *peace of mind*. Moreover, many caregivers recognized the outcomes of this peace of mind, such as better sleep and the ability to take vacations. These observations point to a decrease in caregiver burden, which refers to the often-negative impacts of caregiving on the caregiver’s physical and mental health and overall quality of life [29]. Thus, our findings suggest that the use of CareLink Advantage can provide caregiver relief, which may prevent caregiver burnout and burden and promote less stressful relationships between family or friend caregivers and the older adults they care for.

These findings align with those of Leslie et al [30], who concluded from a series of interviews and surveys with unpaid family caregivers that technology can improve their capacity to provide care to older adults and safeguard their own well-being. Although the evaluation of the impact of passive remote monitoring systems on caregiver burden specifically is yet to be produced, related studies testing assistive technologies to help clients with daily tasks and remote monitoring of vital signs [31] have concluded that their use contributes to reducing caregiver burden.

### Social Workers’ Role

#### Gatekeeping

There seemed to be inconsistency and a lack of standardization regarding social workers’ decisions to recommend the passive remote monitoring system for clients. Although all social workers emphasized the importance of conducting a holistic and comprehensive needs assessment to inform their care plan, the criteria used to determine whether the passive remote monitoring system was a good fit for clients (ie, that the service does a good job meeting the needs of the client) was subjective and varied. Most social workers felt that the passive remote monitoring system would be a good fit for clients in the early stages of dementia who required additional supervision to live safely at home. Others felt that it was not appropriate for older adults prone to wandering or those who did not have a family or friend caregiver who lived close by. This finding is consistent with that of a recent study on home care nurses in Finland [32]. Nurses in this study identified older adults with memory problems as the target group who could benefit most from in-home monitoring. Similar to some of the social workers in our study, these nurses were also worried about wandering behaviors and feared that their clients would wander outside in the middle of the night and get lost, especially because of the long daylight hours in Finland. The similarity of our findings suggests that protecting older adults and ensuring their safety are critical factors influencing care planning decisions when considering the inclusion of in-home passive remote monitoring technologies. Our results also suggest that social workers in the province could benefit from having clear criteria from their needs assessment that would inform their decision of whether to recommend the passive remote monitoring system to their clients and ensuring that all social workers receive training or information about the system as part of their orientation. Finally, it is possible that firsthand experience (or lack thereof) working with clients who had CareLink Advantage may have influenced social workers’ perspectives, resulting in inconsistencies.

#### Role Ambiguity

Role clarity [33,34] ensures that employees know what is expected of them [34]. Role ambiguity occurs when employees do not have a clear understanding of their work roles [35]. Research has shown that professionals’ uncertainness about their roles promotes and aggravates role ambiguity, which can be harmful to everyone [35] and may lead to job burnout and role overload [36]. Our study indicated that social workers were not always sure of their role when it came to informing clients about CareLink Advantage. Decision-makers shared that frontline social workers have very demanding caseloads, resulting in high job demands and a high rate of job turnover. They also felt that these circumstances made it difficult to ensure that all social workers were knowledgeable about all the home support services available to clients. Thus, it makes sense that social workers would default to the services that they were more familiar and comfortable with when discussing home support service options with clients.

#### Perceived Conflicts of Interest

Both social workers and decision-makers identified a perceived conflict of interest for social workers regarding the promotion of the passive remote monitoring system as it was a private business. Interestingly, they did not perceive the same conflict of interest about traditional in-person home support services such as having a personal support worker in the home, although these services are also provided by private businesses. This finding points to a broader discussion about the ethics of the privatization of home support and home care services. As Bjornsdottir [37] explains, there has been a substantial political and policy shift over the past few decades, focused on cost containment (often through strategies such as outsourcing services and rationing care) and increased individual and family responsibilities for home care. Thus, it is interesting that this was only identified as an ethical concern for CareLink Advantage and not for all outsourced services, which also include other technology-based services such as Lifeline. Some strategies that could help alleviate this perceived conflict of interest include having standardized criteria and guidelines for determining which services to recommend to clients based on their needs assessment and using an interprofessional team (eg, occupational therapists and registered nurses) to make the assessment and recommendations for each client.

### Privacy

Our research revealed diverse perspectives regarding the privacy of having the passive remote monitoring system in the home. It was interesting that of all the participant groups, social workers seemed to be the most concerned about the potential for the passive remote monitoring system to invade their clients’ privacy. Clients themselves did not have the same concerns, whereas both caregivers and decision-makers remarked that having numerous personal support workers coming into client homes on a regular basis was more invasive than the remote monitoring technology. Another important finding was that most conversations about privacy were explicitly focused on having a video camera in the home, although this is an optional component.

### Limitations

The findings of this study must be seen considering some limitations. These include: (1) the sample size of clients interviewed—that is, only 2 client participants—which was limited solely to one province, and hence, results may not be applicable to other jurisdictions; (2) the interviews were exclusively conducted in English, which limits the access to other respondents; (3) limited experience of some social workers with the passive remote monitoring system because of low client uptake; and (4) the use of convenience sampling also limits the transferability of our findings to other settings, and hence, the results cannot be treated as representative of the generalized population. A recent study by Young and Casey [38] has shown that samples as small as 6 to 9 participants can provide robust identification of themes and codes in qualitative interview studies. Although our overall sample size was sufficient and the inclusion of multiple perspectives allowed for triangulation, we were unable to reach the minimum sample size for the client group. Therefore, further research is needed on this group to corroborate our initial findings and explore additional perspectives that may not have been included in our study.

Despite these limitations, we believe that the findings emphasize the need for further research on in-home passive remote monitoring technology, which is designed to support aging in place in provinces.

### Recommendations

On the basis of the findings generated from this study, we propose recommendations for practice and research. First, the adoption of in-home monitoring technologies to support aging in place in NB would be better supported by having standardized education and training for frontline social workers about the service and by establishing standardized eligibility criteria for clients. Second, stronger empirical evidence linking in-home technology interventions to staying at home longer and delaying or avoiding institutionalization is needed. We recommend examining these relationships using strong longitudinal research designs, such as randomized controlled trials.

### Conclusions

Our findings show that CareLink Advantage passive remote monitoring is a valuable tool that can provide older adults and their family or friend caregivers in NB with support when it is a good fit for client needs. Key benefits included empowering older adults to stay in their own homes longer and providing caregivers with peace of mind and relief, which improved their quality of life. Our findings also highlight the need to increase public and social workers’ awareness of the service and its benefits. Social workers would also benefit from improved role clarity and more explicit eligibility criteria or guidelines for clients who could benefit from CareLink Advantage.

## Appendix

Multimedia Appendix 1 Summary matrix.

## Abbreviations

NB: New Brunswick
