# Supplementary material for: Stakeholder Perspectives on In-home Passive Remote Monitoring to Support Aging in Place in the Province of New Brunswick, Canada: Rapid Qualitative Investigation
Source: JMIR Aging. 2022 May 11;5(2):e31486. doi: 10.2196/31486 (PMC9133995; doi:10.2196/31486)
Supplement: Multimedia Appendix 1 [file aging_v5i2e31486_app1.docx]

**Multimedia Appendix 1. Summary matrix.**

|  | **Clients**  **(n = 2)** | **Caregivers**  **(n = 11)** | **Social workers**  **(n = 8)** | **Social Development staff**  **(n =7)** |
| --- | --- | --- | --- | --- |
| **Decision to adopt the passive remote monitoring system** | Involved in the decision-making process to adopt the passive remote monitoring system. | Caregiver-led, with input from client and family. | Social workers recommend services based on their client needs assessment; may include the passive remote monitoring system. | Relatively inexpensive process of implementing pilot led by community-based NGO. |
|  | Decision process heavily supported by caregiver and/or social worker | Often in response to changing care needs of client. | Criteria for needing the passive remote monitoring system not standardized. | Social worker recommends the passive remote monitoring system based on their assessment of needs – main concern is the older adults’ safety and security. |
|  |  | Most caregivers were informed by SW, one informed by word of mouth, and another found the technology on her own. | Most social workers recommend the passive remote monitoring system for clients in early stages of dementia who require additional supervision to remain at home. | The complexity of the transition for the caregiver and client influences adoption. |
|  |  | Some clients cognitively unable to participate in the discussion about services. | Provide pamphlet or direct clients/caregivers to the passive remote monitoring system website for information. | Taking on the additional responsibility of new technology can be overwhelming for caregiver. |
|  |  |  | Felt ethical responsibility not to promote private businesses like the passive remote monitoring system. |  |
| **Barriers to adopting the passive remote monitoring system** | Few challenges reported. | Technical setup and infrastructure were initial barriers, but no technical issues after setup. | Not knowing enough about the technology due to lack of promotion. | Social workers taking on an ethical lens, sometimes view discussing the passive remote monitoring system with clients as a conflict of interest. |
|  | Pet rat chewing cords (n =1) | Cost of internet. | Cost to client when not fully subsidized, or the cost of the internet to use the technology. | Workload of SW impedes their ability to learn more about the passive remote monitoring system or take on the responsibility of teaching/guiding caregiver about it. |
|  |  | Learning curve/additional responsibility for caregiver. | Family/client apprehension to use technology. | Client and caregiver apprehension to technology. |
|  |  | Client apprehension to technology. | Geographical location can impact access to internet, power outages. | High turnover rate of social workers creates gaps in knowledge about range of services available. |
|  |  | Increasing care needs of client render the passive remote monitoring system no longer adequate for client needs. | Client uses the passive remote monitoring system as transition when close to need for LTC placement so use may be limited. | Social workers are not introduced to the passive remote monitoring system as part of their orientation (or all services offered/covered). |
|  |  | Pushback from home support services agencies related to privacy concerns. | Family/social worker sees more benefit with in-person care. | Social workers feel they cannot recommend the passive remote monitoring system over another private service. |
|  |  |  | Additional commitment required on the part of the caregiver.  Proximity of client to caregiver. | Biggest barrier is communication to caregiver, difficult to explain to clients. |
|  |  |  | Tech has limits: does not work if caregiver does not use it. | The caregiver is beyond the point of being able to take in a new challenge or responsibility, not able to see benefits. |
|  |  |  | Need to have a caregiver to use it. | Caregiver in crisis does not understand how the passive remote monitoring system can help them. |
|  |  |  | Installation services not offered in both official languages (French and English). | SWs favour in-person care over technology. |
|  |  |  |  | Pride of client – difficult to admit they need help. |
| **Benefits of the passive remote monitoring system** | Increased peace of mind. | Cost-effective alternative to in-home care or residential care. | Cost savings for family and for government in avoiding in-home or residential care. | Main benefit of the passive remote monitoring system is to caregivers who require support. |
|  | Increased accessibility of services. | Reassurance that client/loved one is safer. | The passive remote monitoring system could help meet some of the demands of the current workforce shortage. | Benefits of cost savings to provincial health and long-term care systems. |
|  |  | Client feels safer. | Better relationship between caregiver and client. | Monitoring paid caregivers (abuse, trustworthiness). |
|  | Reduced isolation. | Better sleep for caregiver. |  | Cost savings for family compared to in-person care. |
|  |  | Decreased caregiver burden (participant reported “Peace of mind”); being able to take vacation. |  |  |
|  |  | Caregiver can track care provided by home support workers. |  |  |
| **Impact on health outcomes of client** | Increased accessibility to emergency services when required. | Being able to keep track of evolving care needs. | Clients are able to stay at home longer. | No mechanisms for measuring outcomes of projects like the passive remote monitoring system. |
|  | Being able to stay at home longer. | All caregivers interviewed reported the client they cared for staying at home longer. | Being able to keep track of evolving care needs. | Promotes client choice. |
|  | Peace of mind for client, better sense of security. | No change in health care utilization reported. | There is no process or requirement for social workers to follow-up or collect evaluation data. | Staying at home longer. |
|  |  | Maintain client’s routine. | Institutional placement is often unavoidable when the progression of illness creates a need for supervision and care beyond what the passive remote monitoring system and home support services can offer. |  |
|  |  | Ensure client proper eating |  |  |
|  |  | Adherence/ adjustments to treatment/ medication protocol through observation of changes in behaviour (e.g., increased sleep of older adult) |  |  |
| **Privacy concerns** | Few personal privacy concerns. | Caregivers concerned about the privacy of their parent, but this is outweighed by the additional security and honoring their wish to stay in their own home. | Some concern but not a barrier to recommending the passive remote monitoring system. | Ethical lens taken on by social workers is seen as a barrier to adoption. |
|  | Privacy of caregivers and visitors. | Privacy of paid caregivers who are coming into the home providing services. | Questioning the privacy of caregivers who are not aware of the presence of cameras. | They perceive social worker as not seeing the security vs privacy tradeoff. |
|  |  | Some caregivers reported that clients did not mind the cameras and sensors; did not have objections re: privacy. | Majority of discussion focused on video component of system. | Privacy issues seem to mainly be focused on cameras. |
|  |  | Privacy concerns related to few PSWs working with the older adult in their home; camera was repositioned to minimize PSW exposure. |  | Not necessarily guaranteed privacy with home support workers coming into your home. |
|  |  | At least one participant indicated that technology was less invasive and disruptive than having multiple home support workers entering the home or being in long-term care. |  | General changes in privacy compliance policy have happened since the pilot – the need to keep up with evolving provincial and federal policy on health data security. |
